# Systematic analysis of prognostic and immunologic characteristics associated with coronavirus disease 2019 regulators in acute myeloid leukemia

**DOI:** 10.3389/fgene.2022.959109

**Published:** 2022-09-06

**Authors:** Mingjie Shi, Lidan Chen, Yue Wei, Riling Chen, Runmin Guo, Fei Luo

**Affiliations:** ^1^ Key Laboratory of Research in Maternal and Child Medicine and Birth Defects, Guangdong Medical University, Foshan, China; ^2^ Matenal and Child Research Institute, Shunde Women and Children’s Hospital (Maternity andChild Healthcare Hospital of Shunde Foshan), Guangdong Medical University, Foshan, China; ^3^ First College of Clinical Medicine, Guangdong Medical University, Zhanjiang, China; ^4^ Department of Ultrasound, Shunde Women and Children’s Hospital (Maternity and Child Healthcare Hospital of Shunde Foshan), Guangdong Medical University, Foshan, China; ^5^ Department of Hematology-Oncology, Shunde Women and Children’s Hospital (Maternity and Child Healthcare Hospital of Shunde Foshan), Guangdong Medical University, Foshan, China

**Keywords:** acute myeloid leukemia, COVID-19, differentially expressed genes, prognosis, drug molecule, protein–protein interaction

## Abstract

The coronavirus disease 2019 (COVID-19) pandemic has so far damaged the health of millions and has made the treatment of cancer patients more complicated, and so did acute myeloid leukemia (AML). The current problem is the lack of understanding of their interactions and suggestions of evidence-based guidelines or historical experience for the treatment of such patients. Here, we first identified the COVID-19-related differentially expressed genes (C-DEGs) in AML patients by analyzing RNA-seq from public databases and explored their enrichment pathways and candidate drugs. A total of 76 C-DEGs associated with the progress of AML and COVID-19 infection were ultimately identified, and the functional analysis suggested that there are some shared links between them. Their protein–protein interactions (PPIs) and protein–drug interactions were then recognized by multiple bioinformatics algorithms. Moreover, a COVID-19 gene-associated prognostic model (C-GPM) with riskScore was constructed, patients with a high riskScore had poor survival and apparently immune-activated phenotypes, such as stronger monocyte and neutrophil cell infiltrations and higher immunosuppressants targeting expressions, meaning which may be one of the common denominators between COVID-19 and AML and the reason what complicates the treatment of the latter. Among the study’s drawbacks is that these results relied heavily on publicly available datasets rather than being clinically confirmed. Yet, these findings visualized those C-DEGs’ enrichment pathways and inner associations, and the C-GPM based on them could accurately predict survival outcomes in AML patients, which will be helpful for further optimizing therapies for AML patients with COVID-19 infections.

## Introduction

**FIGURE 1:**
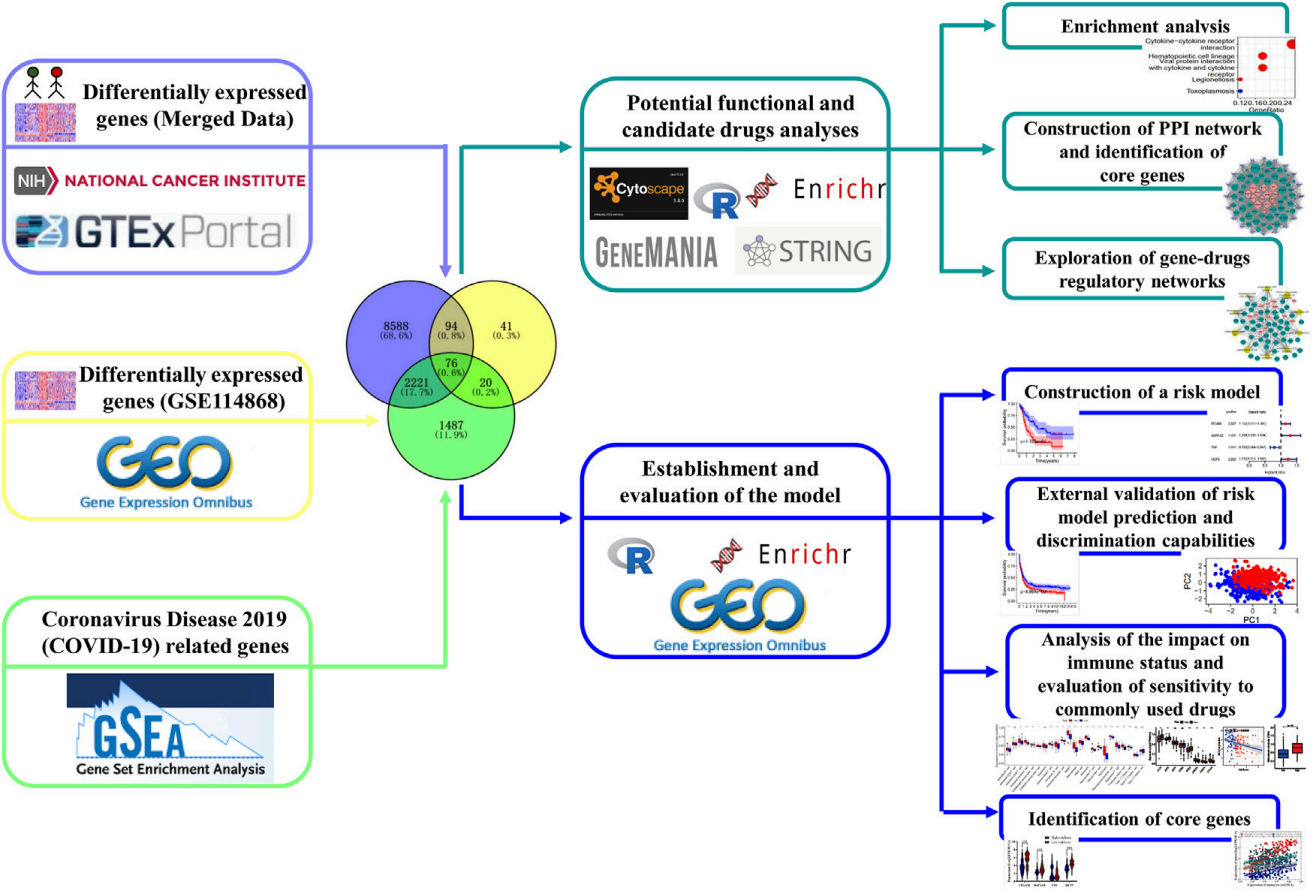
Flow diagram exhibiting the process analysis of the study.

Acute myeloid leukemia (AML) is a common malignancy in adults and is characterized by abnormal proliferation of primitive and naive myeloid cells in the bone marrow and peripheral blood, which has the lowest 5-year survival rate in all leukemia types (Döhner et al., 2015; Westermann and Bullinger 2021). Coronavirus disease 2019 (COVID-19) is an infectious disease caused by the SARS-CoV-2 virus and is mainly manifested by fever, dry cough, fatigue, etc. (Cui et al., 2019; V'Kovski et al., 2021). AML patients have a high risk of getting infected by SARS-CoV-2 owing to their poor resistance and immunity, and the difficulty of treating is undoubtedly greatly increased when AML patients are infected by SARS-CoV-2. Reports on improving the treatment and care of AML patients infected with COVID-19 are also being published (Ferrara et al., 2020; Khan et al., 2020). Farah et al. (2020) established minimal residual disease monitoring in the treatment of NPM1-mutant AML for someone who used updated chemotherapy that had fewer myelosuppressive regimens. Patel et al. (2021) demonstrated that AML patients could activate the immune responses to SARS-CoV-2 even facing immune suppression by chemotherapy. In addition, many studies have previously found that certain gene sets (such as autophagy and immunity) are important in the progression of AML, while the role of COVID-19-related genes in its process is still unknown (Yan et al., 2019; Fu et al., 2021). Thus, identifying the regulatory molecules between them may facilitate providing novel and effective therapeutics for AML patients with COVID-19. In this study, we attempt to identify COVID-19-related differentially expressed genes (C-DEGs), explore their interactions with one another, and discover their candidate drug molecules by multiple bioinformatics. Another prognostic model was constructed through those identified DEGs, and the prognosis performance was validated in the GSE37642 database, and its relationship to the immune microenvironment was also subsequently assessed (Figure 1).

## Materials and methods

### Download and processing of the AML dataset

First, we have comprehensively analyzed the RNA-seq datasets of AML subjects, which were downloaded from The Cancer Genome Atlas (TCGA, https://portal.gdc.cancer.gov/), Genotype-Tissue Expression (GTEx, https://gtexportal.org/home/), and Gene Expression Omnibus (GEO, https://www.ncbi.nlm.nih.gov/geo/) databases. A total of 176 patients with AML in TCGA, 70 normal samples in GTEx, and 194 AML patients and 20 healthy subjects in GSE114868 were used to identify differentially expressed genes. Limma packages with normalizeBetweenArrays function were utilized to merge and emend the data from TCGA and GTEx databases. Additionally, we extracted samples from the clinic in TCGA, according to the following criteria: (a) removing duplicated subjects referred to as formalin-fixed and paraffin-embedded; (b) dislodging subjects with insufficient clinical data; and (c) taking the average of duplicated genes or the same ensemble ID. In total, 152 patients in TCGA and 553 patients in GSE37642 were ultimately incorporated into our study to construct a prognosis model and evaluate its predictive performance (Table 1).

**TABLE 1:** Basic information on datasets in the study.

Series accession number	Platform used	No. of normal samples	No. of tumorous samples	FAB morphology code (%)	Gender (%)	Mean age [min, max]	Vital status (%)	Survive time ( $\bar{x}\pm s$ )
Merge (GTEx and TCGA-LAML)	Illumina RNAseq	70	179 (153 samples with complete clinical data)	M0: 13 (8.5); M1: 34 (22.2); M2: 35 (22.9)	Female: 73 (47.8)	54.2 [18, 88]	Alive: 59 (38.6)	620.8 ± 585.2
				M3: 14 (9.2); M4: 34 (22.2); M5: 17 (11.1); M6:2 (1.3); M7: 3 (2.0)	Male: 80 (52.2)		Dead: 94 (61.4)	
				Unknown: 1 (0.7)				
GSE114868	Affymetrix Human Transcriptome Array 2.0 (GPL17586)	20	194	NA	NA	NA	NA	NA
GSE37642	Affymetrix Human Genome U133A Array (GPL96) and Affymetrix Human Genome U133 Plus 2.0 Array (GPL570)	0	553	M0: 22 (4.0); M1: 113 (20.4); M2: 164 (29.7)	NA	54.9 [18, 85]	Alive: 147 (26.6)	997.0 ± 1,292.5
				M3: 26 (4.7); M4: 121 (21.9); M5: 66 (11.9); M6: 22 (4.0); M7: 3 (0.5)			Dead: 406 (73.4)	
				Unknown: 16 (2.9)				

Abbreviation: FAB, French American British.

### Identification of COVID-19-related differentially expressed genes and functional enrichment analysis

The COVID-19-related gene sets comprising 3,804 genes were downloaded from the Gene Set Enrichment Analysis (GSEA, http://www.gsea-msigdb.org/gsea/index.jsp) database. The Limma package (http://bioconductor.org/packages/release/bioc/html/limma.html) with Benjamini–Hochberg correction and the DESEq2 package (http://bioconductor.org/packages/release/bioc/html/DESeq2.html) were applied to identify differentially expressed genes using Padj <0.001 as all screening criteria. C-DEGs were identified by Venn analysis, and their annotations and functional enrichment analysis on Gene Ontology (GO) and Kyoto Encyclopedia of Genes and Genomes (KEGG) were executed by “clusterProfiler” and “enrichplot” packages, respectively. A *p*-value < 0.05 was deemed as a threshold.

### Protein–protein interaction network analysis and hub genes’ identification

The protein–protein interaction network is composed of proteins that interact with each other to participate in all aspects of biological processes such as signal transmission, gene expression regulation, energy and material metabolism, and cell cycle regulation. Information about the roles of multiple proteins in cells can be integrated into databases and visualized through protein network diagrams. GeneMANIA (http://genemania.org/) (Franz et al., 2018) and STRING (version 11.5, https://string-db.org/) (Szklarczyk et al., 2021) were utilized to explore the PPI networks and hub genes of those identified C-DEGs for further understanding of the physical and functional interactions between AML and COVID-19. All results were visualized by Cytoscape (v.3.7.1, https://cytoscape.org/) (Shannon et al., 2003), which is an open-source network visualization tool to produce an improved performance for different interactions.

### Exploration of candidate drugs

In addition, we also explored the protein–drug interactions or candidate drug molecules based on these identified C-DEGs by the Drug Signatures Database (DSigDB) *via* Enrichr (https://maayanlab.cloud/Enrichr/), and the latter is a web-based comprehensive gene set enrichment analysis tool and can utilize the DSigDB resource to explore related drugs and small molecules.

### Construction of the risk model and analysis of its effect on tumor-infiltrating immune cells and expression of common or emerging immune checkpoints

Samples with completed clinical data in TCGA and GSE37642 databases were applied to construct and validate a prognosis model related to C-DEGs, respectively. Univariate Cox and LASSO regression analyses identified the potential prognosis-associated C-DEGs, and then, multivariate Cox regression analysis was executed to build COVID-19 gene-related prognostic models (C-GPMs) and to assure it was not overfitted. The risk score (riskScore) of each individual was estimated by the following formula: riskScore = [Coefficient 1] ∗ [Expression1] + [Coefficient 2] ∗ [Expression 2] + [Coefficient 3] ∗ [Expression 3] + [Coefficient n] ∗ [Expression n], and the coefficient of each factor was calculated by the LASSO-Cox model. The Kaplan–Meier curve and the receiver operating characteristic curves (ROCs) were utilized to measure the discriminative ability of the C-GPM. In addition, to explore the relationship of this model on the immune microenvironment, the ssGSEA and ESTIMATE algorithms were utilized to calculate the abundance of tumor-infiltrating immune cells (TIICs) and the scores of the tumor microenvironment in each sample, and their correlation and differentiation were separately analyzed by Spearman analysis and Wilcoxon signed-rank test, as they were common or emerging immune checkpoints.

### Cell culture and treatment

The AML cell line KG-1 was provided by Shanghai Yihe Applied Biotechnology Co., Ltd. and was cultured in RPMI-1640 (Gibco, Life Technologies, Carlsbad, CA, USA) that contained 10% fetal bovine serum and 1% penicillin/streptomycin. Cells were grown in a humidified atmosphere with 5% CO2 at 37°C. KG-1 cells were seeded in complete RPMI-1640 medium at appropriate cell numbers and then incubated in the presence of ATRA or RAD001 for the indicated times. RAD001 (everolimus) and all-trans retinoic acid (ATRA) were purchased from APExBIO (Houston, USA) and Sigma Chemical Co. Ltd. (St. Louis, MO), respectively. RNA isolation and real-time PCR were performed based on the corresponding kit instructions.

### Cell counting Kit-8

The CCK8 assay was conducted in accordance with the manufacturer’s instructions (GK10001, GLPBIO). For the assay, 2000 cells/well in 96-well plates containing 100 μL of the culture medium were seeded. A measure of 10 μL of the CCK8 reagent was added to each well at the indicated time, and the plates were given shock for 20 s and then incubated at 37 °C for 2 h. Lastly, we measured the OD value of each hole at 450 nm. These experiments were performed with three replicates, and five parallel samples were measured each time.

### Statistical analysis

Statistical analyses were performed by R software (version: 3.5.2) with multiple packages (including Limma, ggplot2, glmnet, rms, preprocessCore, survminer, and ConsensusClusterPlus) and GraphPad Prism (version 8.4.3, La Jolla, CA, United States) software. Student’s t-test was used to test for significant differences between any two groups of data, and one-way ANOVA was used when evaluating multiple groups of data. All hypothetical tests were two-tailed, and a *p*-value < 0.05 was considered statistically significant.

## Result

### Identification of common DEGs associated with COVID-19 in acute myeloid leukemia and enrichment analysis

**FIGURE 2:**
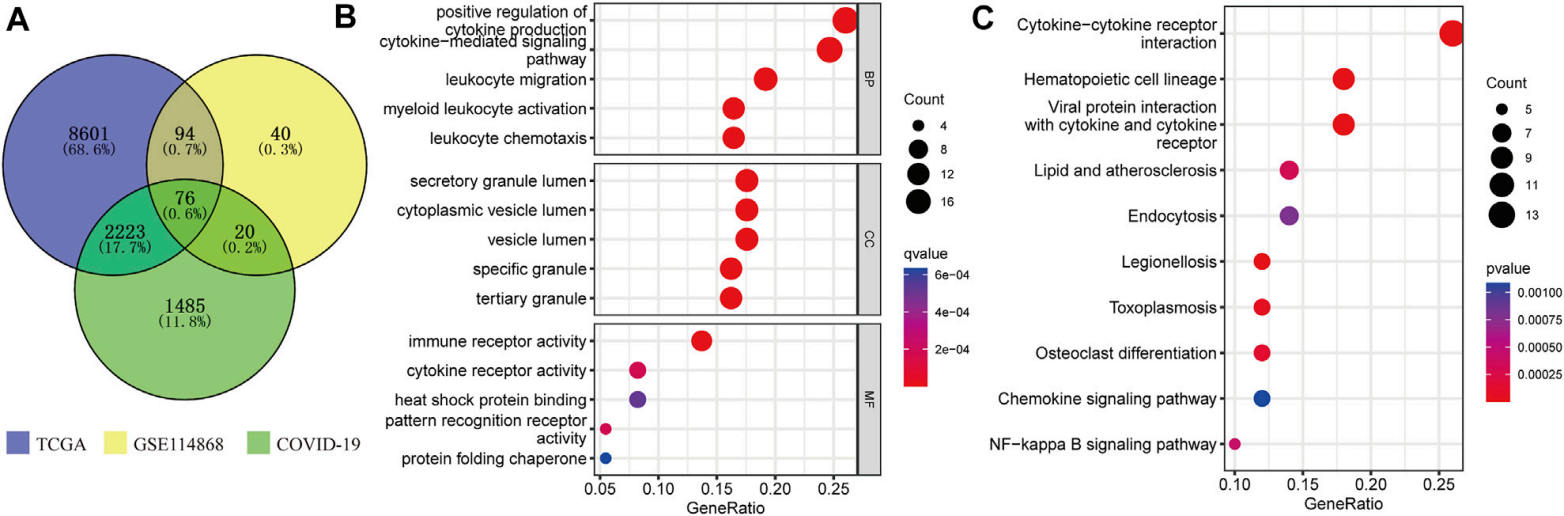
Identification and enrichment analysis of C-DEGs. **(A)** C-DEGs were identified by Venn analysis. GO **(B)** and KEGG **(C)** analyses of those C-DEGs.

The COVID-19-related gene sets comprising 3,804 genes were downloaded from the GSEA database (Supplementary Table S1). A total of 76 C-DEGs were identified with DESeq2 and Limma packages using the adjusted *p*-value < 0.001 as screening criteria (Figure 2A, Supplementary Tables S2 and S3). The top 15 enriched GO terms strikingly exhibited in the bubble chart were intimately concerned with immune inflammation and tumor progressions, such as the positive regulation of cytokine production, the cytokine-mediated signaling pathway, myeloid leukocyte activation, leukocyte chemotaxis, and migration in biological processes (BP); immune, cytokine, and pattern recognition receptors’ activity, heat shock protein binding, and protein folding chaperone in molecular function (MF); and secretory and tertiary granule lumen, cytoplasmic vesicle lumen, and tertiary granule in cellular component (CC) (Figure 2B) (Supplementary Table S4). The KEGG pathways were mainly involved in hematopoietic cell lineage, viral protein interaction with cytokines and cytokine receptors, cytokine–cytokine receptor interaction, and other immune or viral infection-related pathways (Figure 2B) (Supplementary Table S5).

### Construction of the protein–protein interaction network and identification of their candidate drugs

**FIGURE 3:**
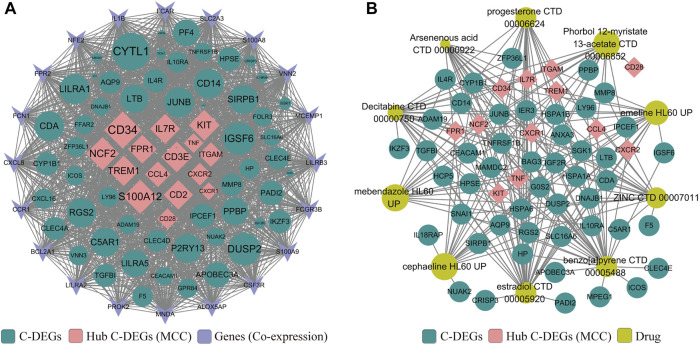
Construction of PPI **(A)** and protein–drug interaction **(B)** networks on those identified C-DEGs by GeneMANIA, STRING, Enrichr, and Cytoscape. Pink represents core C-DEGs that were identified by the MCC algorithm of the Cytoscape plugin (cytoHubba), green represents other C-DEGs, sky blue represents co-expressed genes identified by GeneMANIA, and ginger represents candidate drugs obtained in DSigDB *via* the Enrichr database.

Next, a PPI network was built to systematically analyze the interaction of those C-DEGs in biological systems and understand the response mechanism of biological signals and energy metabolism in special physiological states in-depth, as well as the functional connections among proteins. In our study, PPI networks associated with C-DEGs were constructed through GeneMANIA and STRING tools, and 15 hub signatures (*TNF*, *ITGAM*, *CCL4*, *IL7R*, *CD28*, *CXCR1*, *S100A12*, *CD2*, *TREM1*, *FPR1*, *CD3E*, *CD34*, *NCF2*, *KIT*, and *CXCR2*) were identified based on the network maximal clique centrality (MCC) algorithm of Cytoscape plugin (cytoHubba) (Figure 3A). NetworkAnalyst 3.0 and DrugBank were then employed to explore potential and available drugs targeting these C-DEGs, and a total of 101 drugs were separated using an adjusted *p*-value <0.001 as the threshold (Supplementary Table S6). Here, we visualized 11 of them that targeted more genes, including estradiol, benzo [a]pyrene, decitabine, progesterone, ZINC, cephaeline, arsenenous acid, emetine, mebendazole, and phorbol 12-myristate 13-acetate (Figure 3B).

### Construction and validation of a risk model with four C-DEGs for AML

**FIGURE 4:**
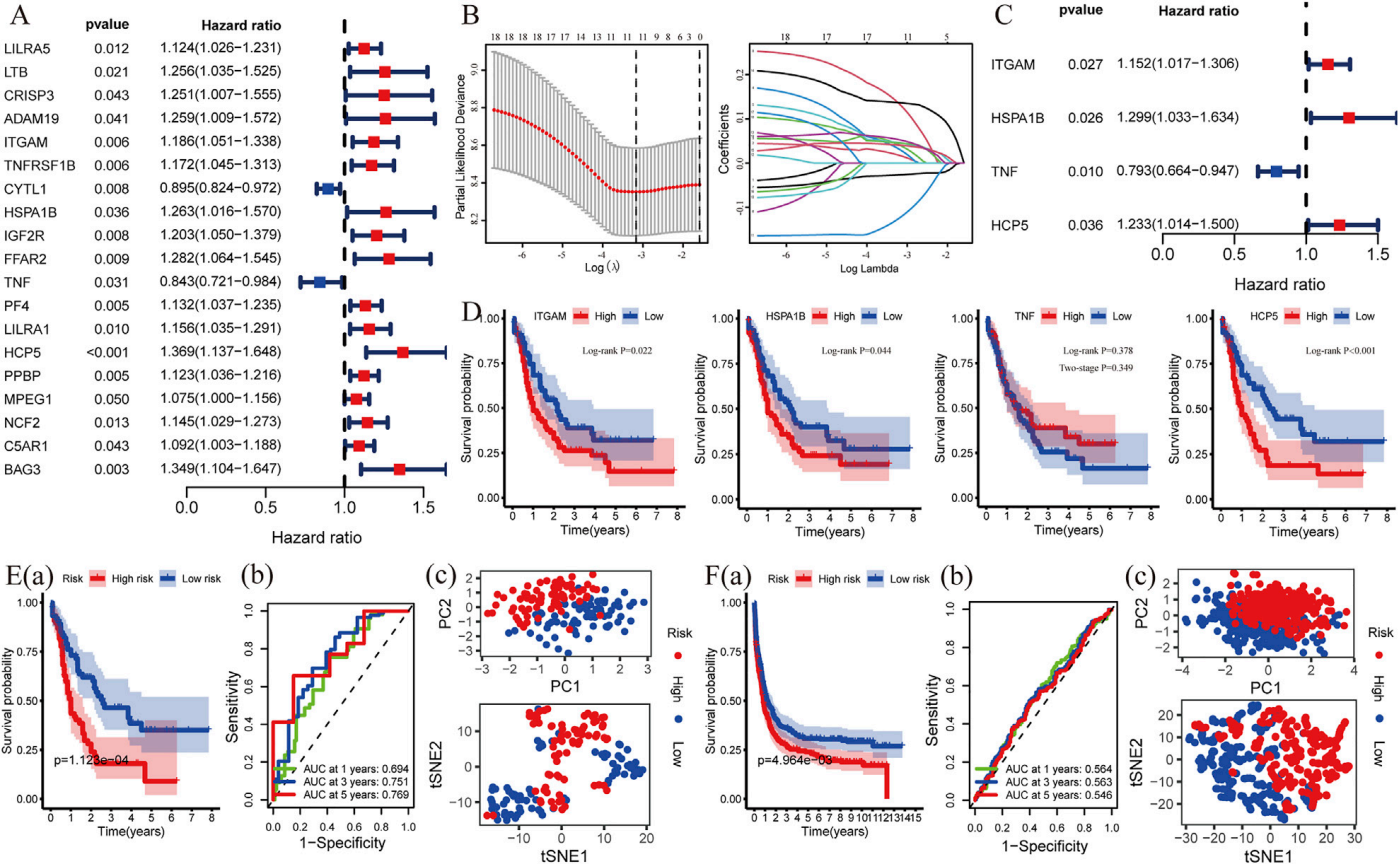
Construction and validation of a risk model associated with identifying C-DEGs. **(A)** Nineteen C-DEGs with prognosis in AML were identified by univariate Cox regression analysis (*p* < 0.05). **(B,C)** LASSO- and multivariate Cox regression analyses were applied to build a risk model (C-GPM), and the forest map exhibited four C-DEGs with their *p* values and hazard ratios (HR) with confidence intervals (CI). **(D)** Kaplan–Meier method was used to calculate their differences in overall survival. **(E)** Predictive and discriminative abilities of the C-GPM in AML were evaluated by multiple methods, including survival- **(a)**, ROC- **(b)**, PCA-, and t-SNE **(c)** analyses. **(F)** Validation of the predictive and discriminative abilities of the C-GPM in the GSE37642 dataset.

To explore whether these C-DEGs are associated with patients’ overall survival, a total of 19 genes were integrated into the Lasso regression analyses after univariate Cox regression (*p*-value < 0.05, Figures 4A,B; Supplementary Table S7). A multivariate Cox proportional hazards regression model was subsequently utilized to construct the C-GPM with riskScore; patients were divided into high- and low-risk groups using the median riskScore as cutoff (Supplementary Table S8). A C-GPM consisting of four genes (*TNF*, *ITGAM*, *HSPA1B*, and *HCP5*) was identified, and they could all serve as independent indices for predicting the patients’ overall survival (Figure 4C). *ITGAM*, *HSPA1B*, and *HCP5* were then confirmed to negatively correlate with the prognosis of AML patients using the Kaplan–Meier method (Figure 4D), and patients with high riskScore had significantly shorter overall survival than those with low riskScore and a favorite prognostic predictive value in determining the survival rates of AML patients (1-year AUC = 0.694, 3-year AUC = 0.751, and 5-year AUC = 0.772; Figure 4E (a) and (b)). Furthermore, the principal component analysis (PCA) and t-distributed random neighbor embedding (t-SNE) analysis showed that the C-GPM could well differentiate patients into two different risk groups (Figure 4Ec). These findings were subsequently validated in the GSE37642 database (Figure 4F).

### Effects of the C-GPM on the immune status and tumor microenvironment

**FIGURE 5:**
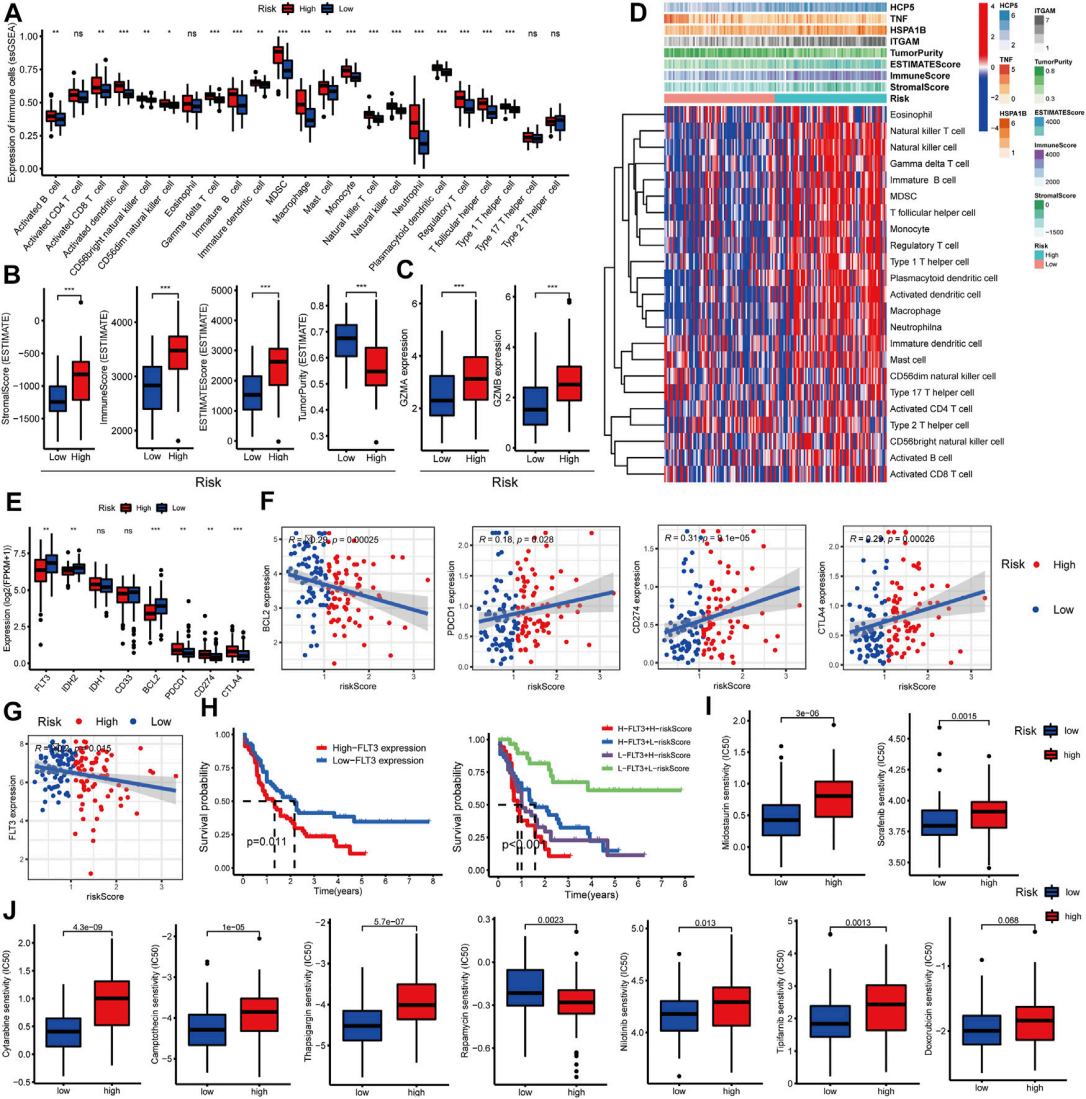
Evaluation of the relationship between the C-GPM and immune microenvironment. **(A,B)** Differences in common TIICs and the tumor microenvironment in the C-GPM were assessed, and the results indicated that patients in the high-risk group had a more pronounced immune or inflammatory activation phenotype. **(C)** Exploration of the difference between GZMA and GZMB that represents immune infiltration and immune cytolytic activity in the C-GPM. **(D)** Heatmap was used to directly show the correlation between the C-GPM with four C-DEGs and the immune microenvironment. **(E,F)** Exploration of whether emerging therapeutic targets are differentially expressed in the two distinct groups of the C-GPM and analysis of their correlation with riskScore. **(G)** Analysis of the correlation between the novel therapeutic target (FLT3) and riskScore. **(H)** Survival analysis of FLT3 with or without riskScore. **(I)** Analysis of the effect of the C-GPM on the sensitivity of midostaurin and sorafenib that modulates the receptor tyrosine kinase FLT3. **(J)** Analysis of the effect of the C-GPM on the sensitivity of other common AML drugs.

In addition, several recent studies have indicated that the abundance of TIICs within the tumor microenvironment (TME) could predict phases of tumor inflammation and were related to the poor prognosis of AML patients. Thus, we explored the impact of the C-GPM on them based on ssGSEA and ESTIMATE algorithms. The cohort was stratified into high riskScore (N = 76) and low riskScore (N = 77) groups according to their medians; most TIICs were more abundant in high-risk groups (Wilcoxon signed-rank test, *p*-value < 0.05, Figure 5A), and stromal (StromalScore), immune (ImmuneScore), and ESTIMATE (ESTIMATEScore) scores were all increased with statistically significant differences in the high-riskScore group, while the tumor purity (TumorPurity) was contrary to their trend (Wilcoxon signed-rank test, *p*-value < 0.05, Figure 5B). Also, we explored the expression levels of granzyme A (GZMA) and granzyme B (GZMB) representing immune infiltration and immune cytolytic activity (Arias et al., 2017). They all showed higher expression in the high-riskScore group, as was expected (Wilcoxon signed-rank test, *p*-value < 0.05, Figure 5C). All these findings suggested that patients with high riskScore had more pronounced immune and inflammatory responses along with more risks and worse prognoses (Figure 5D).

### Analysis of the impact on immunotherapy and evaluation of sensitivity to commonly used drugs

Many explorations have shown that immune checkpoint testing is a reliable way to assess the patient’s response to immunotherapy, which is blossoming into the backbone of cancer treatment, while AML patients with high expression of conventional immune checkpoints [such as programmed cell death 1 (PDCD1, best known as PD1)] did not well benefit from immunotherapy based on most clinical trials, and these are closely related to immune complications in AML patients. In our study, patients with high riskScore had higher expressions of the common immune checkpoints such as PD1, programmed cell death ligand 1 (PDL1/CD274), and cytotoxic T-lymphocyte antigen 4 (CTLA4) and had lower levels of the emerging checkpoints including fms-like tyrosine kinase-3 (FLT3), isocitrate dehydrogenase (NADP (+)) 2 (IDH2), and B-cell leukemia/lymphoma 2 (BCL2) (Figures 5E,F). Additionally, studies have shown that FLT3 is highly expressed in more than 70% of AML patients, and for this, it was considered an important target for the treatment of AML. Here, the FLT3 expression was negatively correlated with riskScore and prognosis of patients with AML; patients with low FLT3 combined with low riskScore had significantly better overall survival than others. Also, those with low riskScore had more sensitivity to drugs, such as midostaurin and sorafenib, that modulate the receptor tyrosine kinase FLT3; the former has been approved as a new treatment option for relapsed or refractory FLT3-mutated AML (Figures 5G–I). In addition, we also explored the effect of C-GPM on the sensitivity of other common AML drugs, and patients in the high-riskScore group were more sensitive and beneficial to cytarabine, camptothecin, thapsigargin, nilotinib, and tipifarnib but were less sensitive to rapamycin. The sensitivity to doxorubicin had no significant difference in both groups (Figure 5J).

### HCP5 might be a novel prognostic immune-related biomarker of AML

**FIGURE 6:**
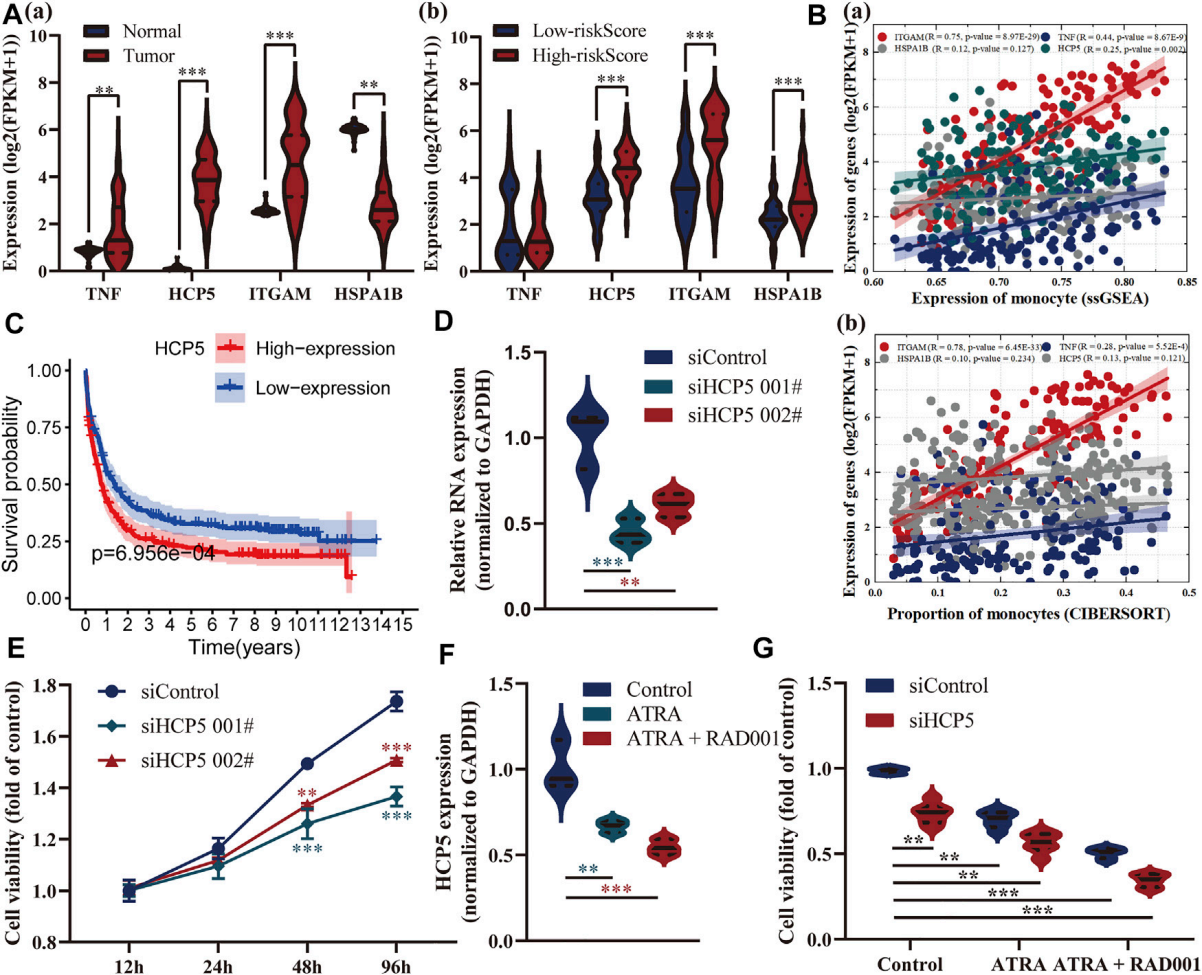
Exploration of the relationship between these genes in the model and AML. **(A)** Analysis of differential expressions of these four genes between AML and healthy patients **(a)**, high-, and low-risk groups **(b)**, respectively. **(B)** Assessment of their correlation with monocytes. **(C)** Survival analysis of HCP5 in the GSE37642 dataset showed that its expression was associated with prognosis. **(D,E)** CCK8 results showed that silencing of HCP5 significantly affected the proliferation of KG-1 cells. **(F,G)** ATRA (1 μM) in combination with RAD001 (10 nM) strikingly downregulated the expression of HCP5 and inhibited cell growth, especially in HCP5-silenced cells.

Also, we delved into the differential expression of these four genes between AML and healthy patients, high-, and low-risk groups, respectively (Figure 6A). HCP5 and ITGAM were both highly expressed in AML patients and high-risk groups, and the latter was considered a marker for monocytes, and a very strong correlation between them was confirmed (Figure 6B). HSPA1B, a receptor that assisted virus entry into cells, is highly expressed in patients in the high-risk group, while TNF has no significant difference in both risk groups. Among them, HCP5 has been shown to have a prognostic role in multiple external datasets, and its high expression is closely associated with poor prognosis in AML (Figure 6C). We subsequently found *in vitro* that silencing of HCP5 significantly affected the proliferation of KG-1 cells derived from the human acute myelogenous leukemia cell line (Figures 6D,E). We identified 413 genes significantly associated with HCP5 through the LinkedOmics database and found that they were mainly associated with immunity in AML patients, including regulation of T-cell activation, lymphocyte-mediated immunity, and Th17 cell differentiation (Supplementary Figure S1). Additionally, all-trans retinoic acid (ATRA) is a traditional drug for the treatment of AML, and everolimus (RAD001) is a new type of immunosuppressant. ATRA (1 μM) in combination with RAD001 (10 nM) strikingly downregulated the expression of HCP5. Notably, RAD001 (10 nM) significantly enhanced the ATRA-inhibited cell growth, and these were more pronounced in HCP5-silenced cells (Figure 6F).

## Discussion

The COVID-19 pandemic has been going on worldwide for over two years; although posing a huge threat to the health of normal people, it also seriously affects the treatment of cancer patients (Henderson et al., 2021). AML is the most common leukemia in adults and accounts for about 80% of all cases, and its treatment and care have been further complicated by the COVID-19 pandemic (Ferrara et al., 2020; Wilde et al., 2020). In this study, we attempted to analyze the potential connections between COVID-19 infections and AML and to explore its impact on prognosis and candidate medications’ susceptibility of AML patients with COVID-19, providing a new insight into their clinical diagnosis and treatment.

Here, we identified 76 C-DEGs in AML, and they were mainly involved in hematopoietic cell lineage, viral protein interaction with cytokines and cytokine receptors, cytokine–cytokine receptor interaction, and other viral infection or tumor progression pathways. In addition, the PPI network constructed by STRING and GeneMANIA has shown that most of them have obvious interactions, and 15 hub C-DEGs (TNF, ITGAM, CCL4, IL7R, CD28, CXCR1, S100A12, CD2, TREM1, FPR1, CD3E, CD34, NCF2, KIT, and CXCR2) were identified and were involved in the maintenance of human homeostasis. Meanwhile, their candidate drugs were also explored, multiple drugs (estradiol, benzo [a]pyrene, decitabine, progesterone, ZINC, cephaeline, arsenenous acid, emetine, mebendazole, and phorbol 12-myristate 13-acetate) were identified, and most have been reported to have antitumor effects. For example, mebendazole has been reported to exhibit potent antileukemic activity against AML, and it is considered to have the potential to bind to SARS-CoV-2 B.1.1.7 (alpha) and P.1 (gamma) variants (He et al., 2018; Yele et al., 2022).

Subsequently, a C-GPM consisting of four genes (*TNF*, *ITGAM*, *HSPA1B*, and *HCP5*) was constructed by multiple analyses, and these genes could serve as independent indices for predicting the patients’ overall survival. TNF, generally known as TNF-α, is mainly secreted by mononuclear macrophages and is a cytokine involved in systemic inflammation. Some studies have reported that TNF can inhibit the replication of different viruses to exert antiviral effects, as well as regulate the function of immunocytes (Liu et al., 1998; Bruunsgaard et al., 2003). Integrin subunit alpha M (ITGAM) is implicated in mediating the uptake of pathogens and in various adhesive interactions of macrophages, monocytes, and granulocytes, and it is also required for CD177-PRTN3-mediated activation of TNF-sensitized neutrophils (Boguslawska et al., 2016; Lyu et al., 2020). Heat shock protein family A (Hsp70) member 1B (HSPA1B) is one of three protein-encoding genes belonging to the HSP70 family and is involved in the human immune response after infection with Epstein–Barr virus, *Legionella*, and influenza A (Sistonen et al., 1994; Soncin et al., 1997; Kiang and Tsokos, 1998). HLA complex P5 (HCP5) is a long non-coding RNA, and emerging studies have recently indicated that it plays an important role in the progression of AML. Research has shown that HCP5 promoted lung adenocarcinoma metastasis *via* the miR-203/SNAI axis and tumor growth and upregulated the expression of PD-L1/CD274 *via* a competing endogenous RNA mechanism of sponging miR-150–5p, and these were also consistent with our findings (Jiang et al., 2019; Xu et al., 2020). The C-GPM could well stratify patients into high- and low-risk groups based on their median riskScores, and patients in the former had worse overall survival, which had also been demonstrated in external cohorts.

Furthermore, we explored the impact of the C-GPM on the immune microenvironment, which was considered a vital criterion in tumor progression and metastasis. The results suggested that patients with high riskScore had more TIICs, higher scores of the tumor microenvironment, and lower tumor purity, implying their immune and inflammatory responses were in a more active state, which increased the difficulty of treatment and the risk of life for AML patients. Notably, patients with high riskScore were shown to have a poor prognosis; this phenomenon may be associated with their immune active status, including immune checkpoints that are highly expressed. Many explorations have shown that immune checkpoint testing is a reliable way to assess the patient’s response to immunotherapy, which is blossoming into the backbone of cancer therapy. Studies show that AML patients with high expression of conventional immune checkpoints (such as *PD1*, *CD274*, and *CTLA4*) did not benefit from immunotherapy, and these are closely related to immune complications (Berger et al., 2008; Chen et al., 2020). In this study, patients with high riskScore had significantly poor prognoses and had obviously high expressions of common immune checkpoints, including *PD1*, *CD274*, and *CTLA4*. With the rapid development of targeted therapy, more and more targets have been discovered with the potential for anticancer, which means that more patients are expected to be covered by targeted therapy drugs. FLT3 is a type III receptor tyrosine kinase (RTK) and plays an important role in the proliferation, differentiation, and survival of hematopoietic stem cells and precursor B cells (Smith et al., 2012; Wu et al., 2018). FLT3 can lead to abnormal cell proliferation and induce tumorigenesis, especially those closely related to the occurrence and development of AML. Studies have shown that FLT3 is highly expressed in more than 70% of AML patients, and for this, FLT3 is considered an emerging important target for the treatment of AML (Gebru and Wang, 2020). Here, the FLT3 expression was negatively correlated with riskScore and prognosis of patients with AML, patients with low FLT3 combined with low riskScore had significantly better overall survival than others, and those with low riskScore had more sensitivity to its inhibitors, such as drugs like sorafenib and the recently approved midostaurin for relapsed or refractory FLT3-mutant AML (Antar et al., 2017; Brinton et al., 2020; Döhner et al., 2020). In addition, we also explored the effect of the C-GPM on the sensitivity of other common AML drugs, and patients in the high-riskScore group were more sensitive and beneficial to cytarabine, camptothecin, thapsigargin, nilotinib, and tipifarnib but were less sensitive to rapamycin. Among them, nilotinib, a second-generation tyrosine kinase inhibitor, is primarily utilized in the treatment of chronic myeloid leukemia and has limited results in the treatment of AML. According to studies, nilotinib has a considerable suppressing effect on CD8^+^ T-lymphocyte activity, which could be one of the reasons why it is more sensitive to patients with a high riskScore (Chen et al., 2008). Tipifarnib is a chemical being explored for the treatment of AML and other types of cancer, and it exhibits substantial immunosuppression. These features may be used as a targeting approach in AML therapy in high-riskScore patients with strong immune activation (Bai et al., 2012; Guo et al., 2020). Additionally, the sensitivity of doxorubicin had no significant difference in both groups; this may be because doxorubicin promotes tumor cell metastasis by releasing inflammatory chemicals, which in turn aggregates monocytes and macrophages and worsens the underlying illness (Keklikoglou et al., 2019). Thapsigargin has antiviral properties and is thought to help curb the spread of epidemics including COVID-19 (Al-Beltagi et al., 2021a; Al-Beltagi et al., 2021b). These results suggest that there are established links between COVID-19 infection and AML progression, and they are related to the overall immune status of patients.

In addition, we did not retrieve reports using public datasets to explore the role of COVID-19-related gene sets in AML patients, although there have been many reports in other tumors. For example, Huang et al. identified a novel prognostic signature and nomogram based on SARS-CoV-2-related genes as reliable prognostic predictors for KIRC patients and provided potential therapeutic targets for KIRC with COVID-19 infection, and Liang et al. revealed commonality in specific gene expression by patients with COVID-19 and LUAD (Huang et al., 2021; Liang et al., 2022). Both COVID-19 and cancers provide complicated challenges that require ongoing research and development in medicine. It is challenging to confirm the results of most research excursions in the clinic because they rely on publicly available datasets, which is one of the primary reasons why these methods have, up to this point, been unable to produce convincing findings. Additionally, the methods by which these putative differential genes contribute to AML development or COVID-19 infection have not been exhaustively investigated.

## Conclusion

AML patients have a high risk of getting infected by SARS-CoV-2 owing to their poor resistance and immunity, so we have analyzed the potential interactions between AML and COVID-19 infection by multiple bioinformatics, such as identifying C-DEGs, exploring their interactions with one another, and discovering candidate drug molecules by the DSigDB database. In addition, a prognostic model with satisfactory prediction performance was constructed through these identified C-DEGs, and patients were divided into high- and low-risk groups with distinct overall survival. We found that patients in the former had poor prognoses and had apparently immune-activated phenotypes, such as more immune cell infiltrations and higher expression of immunosuppressive points. Instead, patients in the latter had more sensitivity to emerging targeted inhibitors, such as midostaurin and sorafenib that modulate the receptor tyrosine kinase FLT3. At present, the number of people infected with SARS-CoV-2 is still increasing sharply; more research studies on the commonality of COVID-19 and other diseases are necessary to provide more treatments for patients.

## Data Availability

Publicly available datasets were analyzed in this study. The publicly available dataset can be downloaded from the UCSC Xena browser (https://gdc.xenahubs.net) and the GEO database (https://www.ncbi.nlm.nih.gov/gds/) under the accession numbers GSE114868 and GSE37642.
