# Supplementary material for: Systematic analysis of prognostic and immunologic characteristics associated with coronavirus disease 2019 regulators in acute myeloid leukemia
Source: Front Genet. 2022 Sep 6;13:959109. doi: 10.3389/fgene.2022.959109 (PMC9485716; doi:10.3389/fgene.2022.959109)
Supplement: Supplementary file 2 [file Image1.pdf]

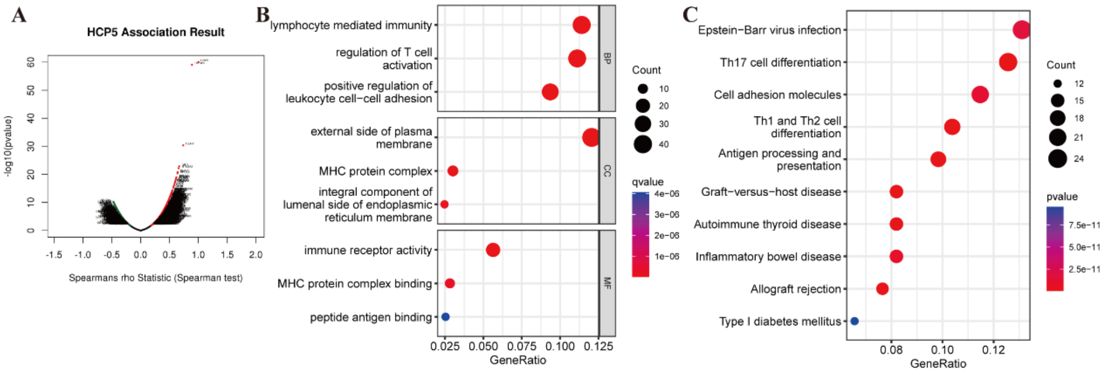

Figure S1. Identification and enrichment analysis of genes associated with HCP5. (A) Genes associated with HCP5 were identified by Linkedomics (http://www.linkedomics.org/admin.php). GO (B) and KEGG (C) analysis of those above genes.
